# New Insights into Molecular Mechanisms Underlying Neurodegenerative Disorders

**DOI:** 10.3390/brainsci12091190

**Published:** 2022-09-03

**Authors:** Chiara Villa, Yam Nath Paudel, Christina Piperi

**Affiliations:** 1 School of Medicine and Surgery, University of Milano-Bicocca, 20900 Monza, Italy; 2 Neuropharmacology Research Laboratory, Jeffrey Cheah School of Medicine and Health Sciences, Monash University Malaysia, Bandar Sunway 47500, Malaysia; 3 Department of Biological Chemistry, Medical School, National and Kapodistrian University of Athens, 11527 Athens, Greece

Neurodegenerative disorders remain a major burden for our society, affecting millions of people worldwide. Due to the symptomatic nature of current treatments without affecting the underlying cause of the disease, these disorders present an ongoing clinical challenge. Extensive research efforts demonstrate the important impact of molecular mechanisms in driving the primary pathological aspects of neurodegenerative diseases and the need for g reater understanding to facilitate targeted drug development.

This Special Issue collects six reviews, five original research articles and one communication in order to provide new insights in the molecular mechanisms that underlie neurodegenerative disorders.

The first entry is an elaborate review of signal transduction mediated by Toll-like receptors (TLRs) on the resident macrophages of the central nervous system (CNS) and neurons bridge immune system response to the pathogenesis of neurodegenerative disorders. A critical evaluation of several studies from animal models and humans highlights the important role of TLR2, TLR3, TLR4, TLR7, and TLR9 in Parkinson’s disease (PD) and Alzheimer’s disease (AD) [1].

In turn, clinical research studies indicate the emerging role of the anti-inflammatory cytokine IL-37 in common CNS diseases. Although IL-37 has been previously associated with the pathogenesis of autoimmune diseases and cancer, the present review by Li et al. focuses on the mechanism of action of IL-37 in CNS and the inhibition of inflammatory cytokines, such as IL-1β, IL-6, and tumor necrosis factor-α (TNF-α), indicating therapeutic potential [2].

In search of the epigenetic mechanisms associated with the pathogenesis of PD, Angelopoulou et al. explored the impact of environmental exposures in altering gene expression [3]. Smoking, coffee consumption, pesticide exposure, and heavy metals (manganese, arsenic, lead, etc.) have been revealed as potential epigenetic modifiers underlying PD development, stimulating the prospects for future research in this direction.

Novel applications of the graph theory and the contribution of electrophysiological techniques in neurodegenerative disorders are further described in the article of Miraglia et al. [4]. They particularly explore the graph theory as an emerging method for the study of functional connectivity in electrophysiological recordings, providing a simple representation of a complex system applied in AD and PD. The benefits of electrophysiological techniques, including their low cost, broad availability, and non-invasive nature, make them potential tools for large population screening.

The potential of the human monoclonal antibody, Aducanumab, as the first approved disease-modifying treatment for AD has been addressed in the article of Gunawardena et al. A critical overview of the promises and controversies associated with Aducanumab in low- and middle-income countries is given, along with contradicting evidence from two clinical trials [5].

As emerging evidence supports the notion that infections by human herpesviruses (HHVs) have been involved in the AD pathogenesis, other authors highlight the current knowledge about the potential molecular interplay between HHVs and AD. In particular, they focus on the main pathological processes of AD, including amyloid beta deposition, tau protein hyperphosphorylation, oxidative stress, autophagy, and neuroinflammation. Therefore, a deeper understanding of these links may help the identification of novel therapeutic targets to prevent or halt the neurodegenerative processes in AD [6].

Moving towards novel research articles, changes in the activity of the rate-limiting enzyme of dopamine synthesis, tyrosine hydroxylase, in the nigrostriatal system of mice has been associated with neurodegeneration and neuroplasticity in an acute model of PD. Detecting differences in the regulation of dopamine synthesis between DA-neuron bodies and their axons can be further considered for the development of symptomatic pharmacotherapy aimed at increasing tyrosine hydroxylase activity [7].

An Italian longitudinal study investigating gut microbiota alterations in fecal samples of PD patients over the period of a year demonstrated stability in microbiota findings. Any differences in the microbiota composition between PD patients and healthy controls also remained stable, without the detection of any worsening in the disease staging or motor impairment in PD patients, paving the way to more extensive longitudinal evaluations [8].

An elegant two-sample Mendelian randomization study with summary statistics from large-scale genome-wide association studies (GWAS) detected a shared genetic background between PD and schizophrenia. Kim et al. evaluated whether genetic variants which increase PD risk influence the risk of developing schizophrenia, and vice versa and detected increased risk of schizophrenia per one-standard deviation (SD) increase in the genetically predicted PD risk. This evidence supports the intrinsic nature of the psychotic symptoms in PD and points out that future studies are needed to investigate possible comorbidities and shared genetic structure between the two diseases [9].

Another intriguing research study employed homology modelling, molecular docking, and molecular dynamics simulation of Calcium homeostasis modulator 1 (CALHM1) in order to test secondary metabolites of *Bauhinia variegata* for AD treatment. Among various flavonoids and alkaloids from *Bauhinia variegata*, quercetin was revealed as a good inhibitor for treating AD, requiring future in vitro and in vivo analyses in order to confirm its effectiveness [10].

A transcriptome sequencing study was performed to screen differentially expressed circular RNAs (DEcircRNAs) in the brains of a rat model of levodopa-induced dyskinesia (LID), a common complication after chronic dopamine-replacement therapy in the treatment of PD. Among a set of 99 DEcircRNAs in the striatum of LID rats, the authors identified high levels of rno-Rsf1_0012 which can competitively bind rno-mir-298-5p, thus abolishing its inhibitory effect on the expression of the target genes, *PCP4* and *TBP*, already associated with other movement disorders. Although these promising results, further investigations are needed to clarify the specific roles of rno-Rsf1_0012 in LID occurrence [11].

Lastly, an important communication proposes a theoretical framework to explain the stochastic processes, at the protein, DNA and RNA levels, which are involved in the development of adult sporadic neurodegenerative disorders [12]. This model of interacting degenerative proteins helps us to elucidate the existence of multiple misfolded proteinopathies in adult sporadic neurodegenerative disorders and may prove highly valuable in the future.

In conclusion, neurodegenerative diseases need integrative understanding of the underlying molecular mechanisms at both the theoretical and practical levels, in order to enable successful clinical management. This Special Issue provides up-to-date information on several aspects of the molecular pathology that underlies some major neurodegenerative disorders, in order to reinforce current thinking and therapeutic approaches.

## Data Availability

Not applicable.
